# 3,3′-Dimethyl-1,1′-(propane-1,3-di­yl)diimidazol-1-ium bis(hexafluoro­phosphate)

**DOI:** 10.1107/S160053680803746X

**Published:** 2008-11-29

**Authors:** Jin-hua Liang, Su-lan Dong, Hui Cang, Jin-tang Wang

**Affiliations:** a Department of Applied Chemistry, College of Science, Nanjing University of Technology, Nanjing 210009, People’s Republic of China; b Huaiyin Institute of Technology, Huaiyin 223003, People’s Republic of China

## Abstract

In the title compound, C_11_H_18_N_4_^2+^·2PF_6_^−^, the dihedral angle between the two planar imidozlium rings is 6.1 (2)°. Both [PF_6_]^−^ anions are disordered [occupancies 0.65 (2):0.35 (2) and 0.59 (5):0.41 (5)]. The crystal packing is stabilized by inter­molecular C—H⋯F hydrogen bonds which link two mol­ecules, forming centrosymmetric dimers.

## Related literature

For applications of dicationic ionic liquids, see: Jared *et al.* (2005 ▶). For bond-length data, see: Allen *et al.* (1987 ▶); Matsumoto & Hagiwara (2007 ▶).
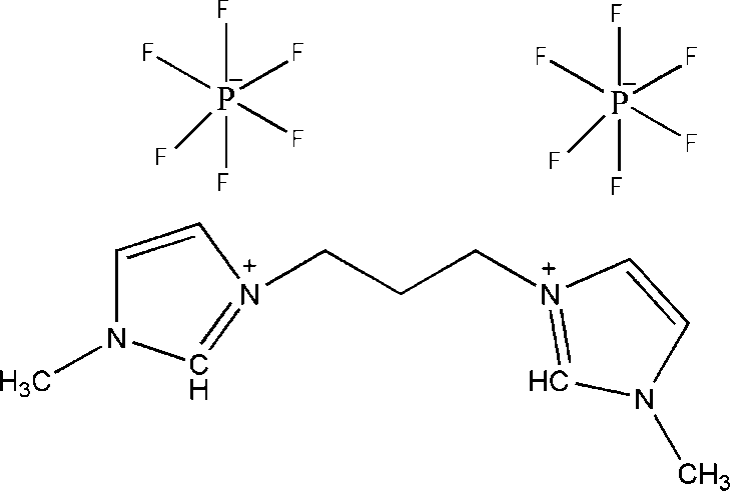


## Experimental

### 

#### Crystal data

C_11_H_18_N_4_^2+^·2PF_6_^−^
*M* *_r_* = 496.23
Triclinic, 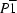

*a* = 8.2300 (16) Å
*b* = 10.192 (2) Å
*c* = 12.856 (3) Å
α = 107.99 (3)°
β = 104.50 (3)°
γ = 96.35 (3)°
*V* = 972.1 (5) Å^3^
*Z* = 2
Mo *K*α radiation
μ = 0.34 mm^−1^
*T* = 298 (2) K
0.30 × 0.30 × 0.20 mm

#### Data collection

Enraf–Nonius CAD-4 diffractometer
Absorption correction: ψ scan (North *et al.*, 1968 ▶) *T*_min_ = 0.905, *T*_max_ = 0.935
3484 measured reflections
3484 independent reflections
2637 reflections with *I* > 2σ(*I*)
3 standard reflections every 200 reflections intensity decay: none

#### Refinement

*R*[*F* ^2^ > 2σ(*F* ^2^)] = 0.065
*wR*(*F* ^2^) = 0.179
*S* = 0.97
3484 reflections
372 parameters
H-atom parameters constrained
Δρ_max_ = 0.30 e Å^−3^
Δρ_min_ = −0.48 e Å^−3^

### 

Data collection: *CAD-4 Software* (Enraf–Nonius, 1989 ▶); cell refinement: *CAD-4 Software*; data reduction: *XCAD4* (Harms & Wocadlo, 1995 ▶); program(s) used to solve structure: *SHELXS97* (Sheldrick, 2008 ▶); program(s) used to refine structure: *SHELXL97* (Sheldrick, 2008 ▶); molecular graphics: *SHELXTL* (Sheldrick, 2008 ▶); software used to prepare material for publication: *SHELXTL*.

## Supplementary Material

Crystal structure: contains datablocks I, global, x1. DOI: 10.1107/S160053680803746X/rn2047sup1.cif

Structure factors: contains datablocks I. DOI: 10.1107/S160053680803746X/rn2047Isup2.hkl

Additional supplementary materials:  crystallographic information; 3D view; checkCIF report

## Figures and Tables

**Table 1:** Hydrogen-bond geometry (Å, °)

*D*—H⋯*A*	*D*—H	H⋯*A*	*D*⋯*A*	*D*—H⋯*A*
C2—H2*A*⋯F6^i^	0.93	2.41	3.256 (16)	151
C7—H7*A*⋯F3	0.97	2.49	3.446 (12)	167

Symmetry code: (i) 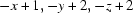.

**Fig. 1.**
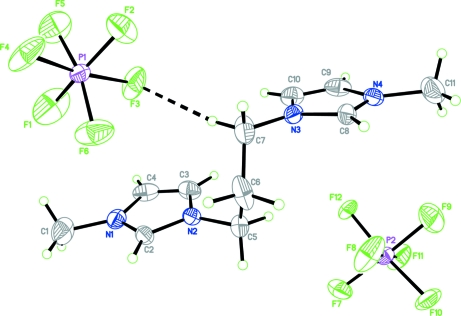
The molecular structure of the title molecule, with the atom-numbering scheme. Displacement ellipsoids are drawn at the 50 % probability level. Dashed lines indicate C-H···F hydrogen bonds.

**Fig. 2.**
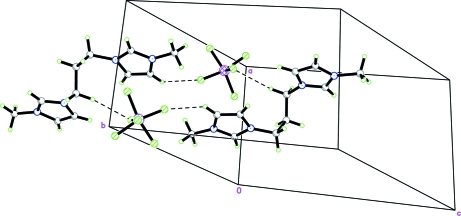
Crystal packing in (I). Dashed lines indicate intermolecular C-H···F hydrogen bonds.

## supplementary crystallographic information

### Comment

The title compound is a dicationic ionic liquid, which has high thermal stability. Applications of the dicationic ionic liquid are found in  biochemistry as well as many areas of chemistry (Jared *et al.*, 2005). We report the crystal structure of the title compound, (I).

In  (I) (Fig. 1), the bond lengths and angles are within normal ranges (Allen *et al.*, 1987). Rings A (C1—C5/N1/N2) and B (C7—C11/N3/N4) are, of course, planar and the dihedral angle between them is 6.1 (2) °.  In the crystal structure, intermolecular C-H···F hydrogen bonds (Table 1) link the molecules (Fig. 2), forming centrosymmetric dimers, which may be effective in the stabilization of the crystals.

### Experimental

1,3-Dibromide propane(10.1 g, 0.05 mol) was added to acetonitrile solution(50 ml) of dehydrate imidazole(9.4 g, 0.11 mol) at 353 K. After stirring for 24 h, the mixture was cooled to room temperature and filtered. The solids were washed with ethyl acetate and dried. Above solid(1.42 g, 5 mmol) was dissolved in distilled water(50 mL) and potassium hexafluorophosphate(1.84 g, 10 mmol) was added. After stirring at room temperature for 3 h, a white solid formed which was collected by filtration, washed with distilled water(20 mL) and dried; The product was purified by repeated crystallization. Crystals of (I) suitable for X-ray diffraction were obtained by slow evaporation of acetone. Each starting material was distilled in advance under reduced pressure with 5 Å molecular sieve. (yield; 0.848 g, 40 %, m.p. 414 K)

### Refinement

Both two distinct hexafluorophosphate groups are disordered over two sites while central P atoms are fixed; the site occupancy factors were refined and converged to 0.65 (2) and 0.35 (2) for F1—F6 and F1'—F6', 0.41 (5) and 0.59 (5) for F7—F12 and F7'—F12' respectively.

H atoms were positioned geometrically, with C—H = 0.93, 0.96 and 0.97 Å for methine, methyl, methylene H, respectively, and constrained to ride on their parent atoms, with *U*_iso_(H) = *xU*_eq_(C), where *x* = 1.5 for methyl H, and *x* = 1.2 for all other H atoms.

### Crystal data

**Table:**

C_11_H_18_N_4_^2+^·2PF_6_^–^	*Z* = 2
*M**_r_* = 496.23	*F*_000_ = 500
Triclinic, *P*1	*D*_x_ = 1.695 Mg m^−^^3^
Hall symbol: -P 1	Melting point: 414 K
*a* = 8.2300 (16) Å	Mo *K*α radiation λ = 0.71073 Å
*b* = 10.192 (2) Å	Cell parameters from 25 reflections
*c* = 12.856 (3) Å	θ = 10–12º
α = 107.99 (3)º	µ = 0.34 mm^−^^1^
β = 104.50 (3)º	*T* = 298 (2) K
γ = 96.35 (3)º	Block, colorless
*V* = 972.1 (5) Å^3^	0.30 × 0.30 × 0.20 mm

### Data collection

**Table:**

Enraf–Nonius CAD-4 diffractometer	*R*_int_ = 0.000
Radiation source: fine-focus sealed tube	θ_max_ = 25.2º
Monochromator: graphite	θ_min_ = 1.8º
*T* = 298(2) K	*h* = −9→9
ω/2θ scans	*k* = −12→11
Absorption correction: ψ scan(North *et al.*, 1968)	*l* = 0→15
*T*_min_ = 0.905, *T*_max_ = 0.935	3 standard reflections
3484 measured reflections	every 200 reflections
3484 independent reflections	intensity decay: none
2637 reflections with *I* > 2σ(*I*)	

### Refinement

**Table:**

Refinement on *F*^2^	Secondary atom site location: difference Fourier map
Least-squares matrix: full	Hydrogen site location: inferred from neighbouring sites
*R*[*F*^2^ > 2σ(*F*^2^)] = 0.065	H-atom parameters constrained
*wR*(*F*^2^) = 0.180	*w* = 1/[σ^2^(*F*_o_^2^) + (0.1017*P*)^2^ + 1.1631*P*] where *P* = (*F*_o_^2^ + 2*F*_c_^2^)/3
*S* = 0.97	(Δ/σ)_max_ = 0.008
3484 reflections	Δρ_max_ = 0.30 e Å^−^^3^
372 parameters	Δρ_min_ = −0.48 e Å^−^^3^
Primary atom site location: structure-invariant direct methods	Extinction correction: none

### Special details

**Table:**

Geometry. All esds (except the esd in the dihedral angle between two l.s. planes) are estimated using the full covariance matrix. The cell esds are taken into account individually in the estimation of esds in distances, angles and torsion angles; correlations between esds in cell parameters are only used when they are defined by crystal symmetry. An approximate (isotropic) treatment of cell esds is used for estimating esds involving l.s. planes.
Refinement. Refinement of *F*^2^ against ALL reflections. The weighted *R*-factor *wR* and goodness of fit *S* are based on *F*^2^, conventional *R*-factors *R* are based on *F*, with *F* set to zero for negative *F*^2^. The threshold expression of *F*^2^ >σ(*F*^2^) is used only for calculating *R*-factors(gt) *etc*. and is not relevant to the choice of reflections for refinement. *R*-factors based on *F*^2^ are statistically about twice as large as those based on *F*, and *R*- factors based on ALL data will be even larger.

### Fractional atomic coordinates and isotropic or equivalent isotropic displacement parameters (Å^2^)

**Table:**

	*x*	*y*	*z*	*U*_iso_*/*U*_eq_	Occ. (<1)
P1	0.17011 (11)	0.74314 (10)	0.95610 (8)	0.0542 (3)	
F1	0.2940 (19)	0.681 (2)	1.0328 (16)	0.131 (5)	0.65 (2)
F2	0.0282 (10)	0.752 (2)	0.8566 (8)	0.109 (4)	0.65 (2)
F3	0.2002 (14)	0.6372 (10)	0.8485 (8)	0.108 (4)	0.65 (2)
F4	0.114 (3)	0.871 (3)	1.042 (3)	0.104 (9)	0.35 (2)
F5	0.0358 (18)	0.6358 (16)	0.9717 (13)	0.105 (4)	0.65 (2)
F6	0.3276 (19)	0.8550 (14)	0.9610 (14)	0.089 (4)	0.65 (2)
F1'	0.309 (3)	0.753 (3)	1.0618 (19)	0.116 (7)	0.35 (2)
F2'	0.072 (3)	0.838 (3)	0.894 (2)	0.107 (6)	0.35 (2)
F3'	0.229 (3)	0.618 (2)	0.880 (3)	0.154 (13)	0.35 (2)
F4'	0.143 (2)	0.8495 (18)	1.0606 (13)	0.115 (5)	0.65 (2)
F5'	0.004 (3)	0.643 (3)	0.937 (3)	0.151 (12)	0.35 (2)
F6'	0.298 (4)	0.843 (3)	0.936 (2)	0.097 (8)	0.35 (2)
P2	0.71510 (11)	0.77369 (9)	0.42397 (8)	0.0508 (3)	
F7	0.863 (3)	0.772 (3)	0.536 (2)	0.073 (5)	0.41 (5)
F8	0.671 (5)	0.902 (2)	0.513 (3)	0.119 (8)	0.41 (5)
F9	0.573 (4)	0.776 (5)	0.328 (3)	0.135 (11)	0.41 (5)
F10	0.803 (4)	0.865 (3)	0.380 (3)	0.070 (4)	0.41 (5)
F11	0.765 (3)	0.6468 (16)	0.3366 (17)	0.084 (5)	0.41 (5)
F12	0.588 (3)	0.659 (2)	0.4499 (13)	0.039 (4)	0.41 (5)
F8'	0.633 (2)	0.8923 (15)	0.4897 (19)	0.088 (4)	0.59 (5)
F9'	0.561 (2)	0.747 (2)	0.3081 (16)	0.080 (4)	0.59 (5)
F7'	0.864 (3)	0.800 (4)	0.526 (2)	0.127 (8)	0.59 (5)
F11'	0.791 (2)	0.6508 (15)	0.359 (2)	0.093 (4)	0.59 (5)
F10'	0.849 (3)	0.891 (2)	0.402 (2)	0.075 (3)	0.59 (5)
F12'	0.602 (3)	0.673 (2)	0.4581 (16)	0.083 (6)	0.59 (5)
N1	0.6733 (4)	0.7139 (4)	0.9682 (3)	0.0524 (8)	
C1	0.7252 (7)	0.7372 (7)	1.0910 (4)	0.0879 (17)	
H1A	0.7686	0.8359	1.1336	0.132*	
H1B	0.8132	0.6858	1.1081	0.132*	
H1C	0.6281	0.7052	1.1119	0.132*	
N2	0.6251 (4)	0.7563 (3)	0.8105 (2)	0.0432 (7)	
C2	0.6823 (4)	0.8143 (4)	0.9236 (3)	0.0452 (8)	
H2A	0.7224	0.9101	0.9650	0.054*	
N3	0.2487 (4)	0.7375 (3)	0.5612 (3)	0.0450 (7)	
C3	0.5781 (5)	0.6144 (4)	0.7827 (4)	0.0597 (11)	
H3A	0.5335	0.5478	0.7091	0.072*	
N4	0.1799 (4)	0.6783 (3)	0.3781 (3)	0.0458 (7)	
C4	0.6078 (5)	0.5889 (4)	0.8803 (4)	0.0645 (12)	
H4A	0.5874	0.5008	0.8873	0.077*	
C5	0.6155 (6)	0.8329 (5)	0.7305 (4)	0.0671 (12)	
H5A	0.5976	0.7665	0.6540	0.081*	
H5B	0.7237	0.8980	0.7523	0.081*	
C6	0.4733 (7)	0.9135 (5)	0.7285 (4)	0.0737 (14)	
H6A	0.4898	0.9764	0.8060	0.088*	
H6B	0.4830	0.9715	0.6822	0.088*	
C7	0.2951 (6)	0.8265 (5)	0.6833 (4)	0.0699 (13)	
H7A	0.2848	0.7666	0.7281	0.084*	
H7B	0.2147	0.8886	0.6931	0.084*	
C8	0.2296 (4)	0.7848 (4)	0.4749 (3)	0.0440 (8)	
H8A	0.2486	0.8793	0.4819	0.053*	
C9	0.1662 (5)	0.5574 (4)	0.4025 (4)	0.0606 (11)	
H9A	0.1337	0.4660	0.3495	0.073*	
C10	0.2074 (5)	0.5934 (4)	0.5150 (4)	0.0609 (11)	
H10A	0.2082	0.5320	0.5556	0.073*	
C11	0.1433 (6)	0.6887 (6)	0.2638 (4)	0.0789 (14)	
H11A	0.1623	0.7862	0.2709	0.118*	
H11B	0.0261	0.6447	0.2209	0.118*	
H11C	0.2176	0.6422	0.2247	0.118*	

### Atomic displacement parameters (Å^2^)

**Table:**

	*U*^11^	*U*^22^	*U*^33^	*U*^12^	*U*^13^	*U*^23^
P1	0.0474 (5)	0.0577 (6)	0.0566 (6)	0.0048 (4)	0.0160 (4)	0.0139 (4)
F1	0.124 (8)	0.185 (12)	0.171 (12)	0.084 (8)	0.069 (8)	0.146 (11)
F2	0.081 (4)	0.157 (10)	0.082 (5)	0.029 (6)	0.005 (3)	0.047 (6)
F3	0.141 (7)	0.077 (5)	0.081 (5)	−0.021 (5)	0.066 (4)	−0.019 (3)
F4	0.098 (10)	0.049 (6)	0.17 (2)	0.013 (6)	0.100 (12)	−0.003 (9)
F5	0.107 (9)	0.090 (6)	0.131 (6)	−0.011 (5)	0.063 (6)	0.047 (5)
F6	0.068 (4)	0.059 (4)	0.130 (10)	−0.007 (3)	0.047 (5)	0.013 (5)
F1'	0.073 (7)	0.159 (18)	0.093 (8)	0.019 (11)	−0.019 (7)	0.050 (12)
F2'	0.091 (11)	0.111 (12)	0.139 (15)	0.061 (10)	0.019 (10)	0.070 (11)
F3'	0.174 (16)	0.104 (11)	0.12 (3)	−0.081 (12)	0.09 (2)	−0.047 (15)
F4'	0.174 (11)	0.088 (8)	0.079 (5)	0.007 (6)	0.077 (6)	0.000 (5)
F5'	0.060 (7)	0.090 (13)	0.24 (3)	−0.031 (7)	0.080 (13)	−0.039 (14)
F6'	0.131 (18)	0.096 (11)	0.057 (7)	−0.035 (9)	0.054 (9)	0.017 (6)
P2	0.0517 (5)	0.0472 (5)	0.0584 (6)	0.0079 (4)	0.0169 (4)	0.0189 (4)
F7	0.055 (6)	0.096 (8)	0.063 (7)	0.011 (5)	−0.004 (5)	0.040 (7)
F8	0.23 (2)	0.039 (6)	0.108 (10)	−0.009 (9)	0.120 (12)	0.005 (6)
F9	0.093 (12)	0.21 (3)	0.128 (19)	0.047 (15)	0.005 (11)	0.12 (2)
F10	0.063 (10)	0.053 (10)	0.089 (9)	−0.027 (6)	0.026 (8)	0.027 (8)
F11	0.129 (11)	0.052 (7)	0.060 (7)	−0.011 (8)	0.058 (6)	−0.008 (6)
F12	0.049 (6)	0.027 (5)	0.041 (6)	−0.003 (4)	0.021 (4)	0.011 (4)
F8'	0.109 (7)	0.042 (5)	0.144 (11)	0.041 (5)	0.087 (6)	0.029 (6)
F9'	0.059 (5)	0.120 (7)	0.059 (5)	0.015 (4)	0.008 (3)	0.036 (5)
F7'	0.081 (7)	0.191 (17)	0.104 (8)	−0.001 (9)	−0.013 (5)	0.084 (9)
F11'	0.098 (6)	0.084 (7)	0.157 (12)	0.063 (6)	0.096 (7)	0.063 (8)
F10'	0.068 (8)	0.046 (6)	0.103 (9)	−0.028 (5)	0.044 (7)	0.017 (5)
F12'	0.085 (9)	0.066 (9)	0.110 (10)	0.005 (6)	0.055 (8)	0.032 (7)
N1	0.0406 (17)	0.073 (2)	0.0564 (19)	0.0199 (15)	0.0175 (15)	0.0355 (18)
C1	0.074 (3)	0.144 (5)	0.075 (3)	0.039 (3)	0.024 (3)	0.072 (4)
N2	0.0390 (15)	0.0474 (17)	0.0381 (15)	0.0049 (12)	0.0096 (12)	0.0108 (13)
C2	0.0433 (19)	0.047 (2)	0.0394 (19)	0.0078 (15)	0.0073 (15)	0.0113 (16)
N3	0.0434 (16)	0.0528 (18)	0.0523 (18)	0.0164 (13)	0.0173 (14)	0.0321 (15)
C3	0.052 (2)	0.046 (2)	0.065 (3)	0.0067 (17)	0.013 (2)	0.0041 (19)
N4	0.0344 (15)	0.0536 (18)	0.0444 (17)	0.0071 (13)	0.0079 (12)	0.0139 (14)
C4	0.050 (2)	0.050 (2)	0.101 (4)	0.0120 (18)	0.024 (2)	0.033 (2)
C5	0.065 (3)	0.085 (3)	0.047 (2)	−0.006 (2)	0.010 (2)	0.030 (2)
C6	0.111 (4)	0.059 (3)	0.039 (2)	0.019 (3)	0.002 (2)	0.0170 (19)
C7	0.081 (3)	0.097 (4)	0.050 (2)	0.047 (3)	0.026 (2)	0.035 (2)
C8	0.0453 (19)	0.0414 (19)	0.051 (2)	0.0084 (15)	0.0115 (16)	0.0261 (17)
C9	0.044 (2)	0.037 (2)	0.089 (3)	0.0003 (16)	0.012 (2)	0.014 (2)
C10	0.048 (2)	0.054 (2)	0.094 (3)	0.0080 (18)	0.018 (2)	0.049 (2)
C11	0.068 (3)	0.108 (4)	0.053 (3)	0.020 (3)	0.009 (2)	0.024 (3)

### Geometric parameters (Å, °)

**Table:**

P1—F2	1.536 (8)	C1—H1C	0.9600
P1—F5'	1.534 (19)	N2—C2	1.322 (4)
P1—F4'	1.528 (12)	N2—C3	1.362 (5)
P1—F6'	1.51 (2)	N2—C5	1.465 (5)
P1—F1	1.563 (10)	C2—H2A	0.9300
P1—F1'	1.509 (18)	N3—C8	1.322 (4)
P1—F3'	1.556 (16)	N3—C10	1.369 (5)
P1—F5	1.561 (11)	N3—C7	1.475 (5)
P1—F3	1.565 (7)	C3—C4	1.328 (6)
P1—F2'	1.601 (12)	C3—H3A	0.9300
P1—F6	1.603 (14)	N4—C8	1.307 (5)
P1—F4	1.62 (2)	N4—C9	1.362 (5)
F1'—F4'	1.77 (3)	N4—C11	1.463 (5)
P2—F11'	1.565 (10)	C4—H4A	0.9300
P2—F7'	1.48 (2)	C5—C6	1.501 (7)
P2—F12'	1.54 (2)	C5—H5A	0.9700
P2—F9	1.48 (3)	C5—H5B	0.9700
P2—F7	1.65 (3)	C6—C7	1.497 (7)
P2—F10	1.44 (3)	C6—H6A	0.9700
P2—F8'	1.572 (9)	C6—H6B	0.9700
P2—F10'	1.674 (19)	C7—H7A	0.9700
P2—F8	1.596 (18)	C7—H7B	0.9700
P2—F12	1.65 (2)	C8—H8A	0.9300
P2—F11	1.597 (13)	C9—C10	1.319 (6)
P2—F9'	1.62 (2)	C9—H9A	0.9300
N1—C2	1.323 (5)	C10—H10A	0.9300
N1—C4	1.358 (6)	C11—H11A	0.9600
N1—C1	1.463 (5)	C11—H11B	0.9600
C1—H1A	0.9600	C11—H11C	0.9600
C1—H1B	0.9600		
			
F2—P1—F5'	70.4 (13)	F10'—P2—F9'	95.3 (12)
F5'—P1—F4'	90.8 (12)	C2—N1—C4	107.8 (3)
F5'—P1—F6'	160 (2)	C2—N1—C1	124.8 (4)
F4'—P1—F6'	97.2 (11)	C4—N1—C1	127.3 (4)
F2—P1—F1	160.9 (14)	N1—C1—H1A	109.5
F5'—P1—F1'	112 (2)	N1—C1—H1B	109.5
F4'—P1—F1'	71.3 (12)	H1A—C1—H1B	109.5
F6'—P1—F1'	87.2 (14)	N1—C1—H1C	109.5
F5'—P1—F3'	89.3 (13)	H1A—C1—H1C	109.5
F4'—P1—F3'	161.5 (16)	H1B—C1—H1C	109.5
F6'—P1—F3'	88.8 (14)	C2—N2—C3	108.1 (3)
F1'—P1—F3'	91.6 (14)	C2—N2—C5	125.3 (3)
F2—P1—F5	88.4 (7)	C3—N2—C5	126.6 (3)
F1—P1—F5	80.4 (9)	N1—C2—N2	108.9 (3)
F2—P1—F3	77.4 (8)	N1—C2—H2A	125.6
F1—P1—F3	88.5 (7)	N2—C2—H2A	125.6
F5—P1—F3	96.3 (7)	C8—N3—C10	107.2 (3)
F5'—P1—F2'	93.1 (12)	C8—N3—C7	125.2 (3)
F4'—P1—F2'	81.4 (13)	C10—N3—C7	127.5 (3)
F6'—P1—F2'	70.4 (15)	C4—C3—N2	107.2 (4)
F1'—P1—F2'	142 (2)	C4—C3—H3A	126.4
F3'—P1—F2'	117 (2)	N2—C3—H3A	126.4
F2—P1—F6	101.0 (9)	C8—N4—C9	108.1 (3)
F1—P1—F6	91.1 (7)	C8—N4—C11	125.4 (4)
F5—P1—F6	170.3 (9)	C9—N4—C11	126.5 (4)
F3—P1—F6	88.2 (6)	C3—C4—N1	108.0 (4)
F2—P1—F4	88.3 (15)	C3—C4—H4A	126.0
F1—P1—F4	106.7 (12)	N1—C4—H4A	126.0
F5—P1—F4	89.2 (10)	N2—C5—C6	112.3 (4)
F3—P1—F4	164.5 (13)	N2—C5—H5A	109.2
F6—P1—F4	88.6 (9)	C6—C5—H5A	109.2
P1—F1'—F4'	54.9 (9)	N2—C5—H5B	109.1
P1—F4'—F1'	53.8 (8)	C6—C5—H5B	109.1
F11'—P2—F7'	87.0 (11)	H5A—C5—H5B	107.9
F11'—P2—F12'	92.0 (9)	C7—C6—C5	115.9 (4)
F7'—P2—F12'	93.3 (13)	C7—C6—H6A	108.3
F9—P2—F7	175.4 (16)	C5—C6—H6A	108.3
F9—P2—F10	79 (2)	C7—C6—H6B	108.3
F7—P2—F10	104.1 (17)	C5—C6—H6B	108.3
F11'—P2—F8'	177.5 (8)	H6A—C6—H6B	107.4
F7'—P2—F8'	93.5 (11)	N3—C7—C6	113.1 (4)
F12'—P2—F8'	85.5 (9)	N3—C7—H7A	109.0
F11'—P2—F10'	90.5 (10)	C6—C7—H7A	109.0
F7'—P2—F10'	81.4 (19)	N3—C7—H7B	109.0
F12'—P2—F10'	174.0 (13)	C6—C7—H7B	109.0
F8'—P2—F10'	92.0 (10)	H7A—C7—H7B	107.8
F9—P2—F8	91.7 (16)	N4—C8—N3	109.4 (3)
F7—P2—F8	85.2 (16)	N4—C8—H8A	125.3
F10—P2—F8	92.9 (14)	N3—C8—H8A	125.3
F9—P2—F12	90.7 (15)	C10—C9—N4	107.6 (4)
F7—P2—F12	86.0 (11)	C10—C9—H9A	126.2
F10—P2—F12	169.4 (14)	N4—C9—H9A	126.2
F8—P2—F12	91.3 (10)	C9—C10—N3	107.6 (3)
F9—P2—F11	89.6 (16)	C9—C10—H10A	126.2
F7—P2—F11	93.5 (12)	N3—C10—H10A	126.2
F10—P2—F11	86.4 (15)	N4—C11—H11A	109.5
F8—P2—F11	178.3 (13)	N4—C11—H11B	109.5
F12—P2—F11	89.8 (9)	H11A—C11—H11B	109.5
F11'—P2—F9'	91.1 (10)	N4—C11—H11C	109.5
F7'—P2—F9'	176.2 (13)	H11A—C11—H11C	109.5
F12'—P2—F9'	90.1 (10)	H11B—C11—H11C	109.5
F8'—P2—F9'	88.5 (10)		

### Hydrogen-bond geometry (Å, °)

**Table:**

*D*—H···*A*	*D*—H	H···*A*	*D*···*A*	*D*—H···*A*
C2—H2A···F6^i^	0.93	2.41	3.256 (16)	151
C7—H7A···F3	0.97	2.49	3.446 (12)	167

Symmetry codes: (i) −*x*+1, −*y*+2, −*z*+2.
